# Supplementary material for: Environmental and Food Safety Assessment of Pre-Harvest Activities in Local Small-Scale Fruit and Vegetable Farms in Northwest Portugal: Hazard Identification and Compliance with Good Agricultural Practices (GAPs)
Source: Foods. 2025 Jun 18;14(12):2129. doi: 10.3390/foods14122129 (PMC12191982; doi:10.3390/foods14122129)
Supplement: Supplementary file 1 [file foods-14-02129-s001.zip › foods-3624565-supplementary.pdf]

Supplementary material

Table S1

Analytical performance of the applied methodology for the analysis of agrochemicals in all the samples.

Family group	Active compound	Linearity (R^2)	RSD (%)	LOD (µg/L)	LOQ (µg/L)	LOD (µg/kg)	LOQ (µg/kg)
OCPs	α-hexachlorocyclohexanes (α-HCH)	0.9983	4.7	5.76	19.19	0.17	0.58
	β-hexachlorocyclohexanes (β-HCH)	0.9960	8.6	11.47	38.22	0.53	1.76
	Hexachlorobenzene (HCB)	0.9993	4.1	9.77	32.56	0.29	0.98
	[1,1,1 trichloro-2,2-bis- (p-chlorophenyl) ethane] (o,p'-DDT)	0.9964	7.0	14.51	48.35	0.44	1.45
	[2,2-bis(p-chlorophenyl)-1,1-dichloroethylene] (p,p'-DDE)	0.9961	7.1	9.17	30.57	0.49	1.64
	1-chloro-4-[2,2-dichloro-1-(4-chlorophenyl)ethyl]benzene (p,p'-DDD)	0.9999	2.8	5.27	17.56	0.16	0.53
	Aldrin	0.9950	8.4	20.32	67.73	0.63	2.09
	Dieldrin	0.9994	3.7	8.63	28.77	0.26	0.86
	Endosulfan I	0.9991	4.6	10.90	36.34	0.33	1.09
OPPs	Chlorfenvinphos	0.9993	5.6	7.38	24.59	0.22	0.74
	Chlorpyrifos	1.0000	0.9	1.19	3.96	0.04	0.12
	Chlorpyrifos-methyl	0.9998	2.8	3.66	12.21	0.11	0.37
	Malathion	0.9965	9.5	15.65	52.16	0.47	1.56
	Dimethoate	0.9996	4.3	5.62	18.75	0.17	0.56
Pyrethroids	Bifentrin	0.9988	5.1	9.18	30.59	0.28	0.92
	Cyhalothrin I	0,9966	5.0	11.28	37.59	0.34	1.13
	Cyhalothrin II	0.9987	5.8	11.59	38.64	0.35	1.16
	Cypermethrin	0.9958	6.6	15.23	50.77	0.46	1.52

	Deltamethrin I	0.9986	3.8	8.74	29.13	0.26	0.87
	Deltamethrin II	0.9979	4.6	10.74	35.81	0.32	1.07
	Fenvalerate I	0.9956	7.9	19.61	65.36	0.59	1.96
	Fenvalerate II	0.9986	5.6	13.16	43.85	0.39	1.32

Table S2

Analytical performance of the applied methodology for the analysis of flame retardants and PCBs in all the samples.

Compound group	Compound	Linearity (R^2)	RSD (%)	LOD (µg/L)	LOQ (µg/L)	LOD (µg/kg)	LOQ (µg/kg)
Polybrominated diphenyl ethers (PBDEs)	2,4,4’-tribromodiphenyl ether (BDE 28)	0.9984	9.5	18.07	60.23	0.54	1.81
	2,2’,4,4’-Tetrabromodiphenyl ether (BDE 47)	0.9991	3.4	7.00	23.32	0.21	0.70
	2,2’,4,4’,5-Pentabromodiphenyl ether (BDE 99)	0.9990	4.8	11.42	38.07	0.34	1.14
	2,2’,4,4’,6’-Hexabromodiphenyl ether (BDE 100)	0.9982	6.4	15.01	50.04	0.45	1.50
	2,2’,4,4’,5,5’-Hexabromodiphenyl ether (BDE 153)	0.9954	7.4	11.75	39.17	0.35	1.18
	2,2’,4,4’,5,6’-hexabromodiphenyl ether (BDE154)	0.9973	5.3	12.16	40.55	0.36	1.22
	2,2’,3,4,4’,5’,6-Heptabromodiphenyl ether (BDE 183)	0.9975	5.4	8.59	28.64	0.26	0.86
Polychlorinated biphenyls (PCBs)	2,4,4'-Trichlorobiphenyl (PCB 28)	0.9951	8.0	18.53	61.78	0.56	1.85
	2,3',4,4',5-Pentachlorobiphenyl (PCB 118)	0.9963	9.2	21.74	72.47	0.65	2.17
	2,2',4,4',5,5'-Hexachlorobiphenyl (PCB 153)	0.9954	6.3	19.00	63.33	0.57	1.90
	2,2',3,4,4',5,5'-Heptachlorobiphenyl (PCB 180)	0.9961	9.0	17.84	59.46	0.54	1.78
	Tris (2-butoxyehyl) phosphate (TBEP)	0.9971	11.6	15.24	50.79	0.46	1.52
Phosphorus flame retardants (PFRs)	Tris(2-chloroethyl) phosphate (TCEP)	0.9992	4.7	6.36	21.18	0.19	0.64
	Tri-cresyl phosphate I isomer (TCP I)	0.9985	5.5	8.91	29.71	0.27	0.89
	Tri-cresyl phosphate II isomer (TCP II)	0.9979	8.36	12.53	41.78	0.38	1.25

Tri-cresyl phosphate III isomer (TCP III)	0.9953	11.83	15.97	53.24	0.48	1.60
Tris(2-ethylhexyl) phosphate (TEHP)	0.9990	5.3	7.16	23.87	0.21	0.72
Tri-iso-butyl phosphate (TiBP)	0.9957	6.8	10.20	34.01	0.46	1.52
Tri-n-butyl phosphate (TnBP)	0.9996	3.3	4.40	14.67	0.13	0.44
Triphenyl phosphate (TPhP)	0.9997	1.8	3.64	12.13	0.11	0.36
Tripropyl phosphate (TPrP)	0.9971	6.8	16.96	56.52	0.51	1.70

Table S3

Analytical performance of the applied methodology for the analysis of nitrate in all the samples.

Linearity (R^2)	RSD (%)	LOD (mg/L)	LOQ (mg/L)	Sample	LOD (mg/kg)	LOQ (mg/kg)
0.9999	0.79	0.39	1.30	Food	2.59	8.62
				Soil	12.94	43.12

Table S4

Analytical performance of the applied methodology for the analysis of heavy metals in food samples.

Type of sample	Farmer ID	Heavy metals (mg/kg fresh weight for food)													
		Zn		Cd		Pb		Ni		Cu		Cr		Hg	
		LOD	LOQ	LOD	LOQ	LOD	LOQ	LOD	LOQ	LOD	LOQ	LOD	LOQ	LOD	LOQ
Apple (*Malus domestica*)	BB	1.138	3.793	0.006	0.020	0.035	0.117	0.017	0.055	0.167	0.557	0.431	1.438	0.010	0.031
	GP	1.655	5.518	0.009	0.029	0.051	0.170	0.024	0.080	0.243	0.811	0.627	2.091	0.015	0.046
Bell pepper (*Capsicum annuum*)	CB	0.005	0.015	0.027	0.089	0.871	2.905	0.013	0.042	0.128	0.427	0.330	1.101	0.008	0.024
	EB	0.794	2.646	0.004	0.014	0.024	0.081	0.012	0.038	0.117	0.389	0.301	1.003	0.007	0.022

	IP	0.528	1.759	0.003	0.009	0.016	0.054	0.008	0.026	0.078	0.258	0.200	0.667	0.005	0.015
	JB	0.444	1.479	0.002	0.008	0.014	0.045	0.006	0.021	0.065	0.217	0.168	0.561	0.004	0.012
	QB	0.820	2.732	0.004	0.014	0.025	0.084	0.012	0.040	0.120	0.402	0.311	1.036	0.008	0.023
Bok choy (*Brassica rapa*)	KP	0.902	3.007	0.005	0.016	0.028	0.092	0.013	0.044	0.133	0.442	0.342	1.140	0.008	0.025
Carrots (*Daucus carota*)	LP	0.006	0.022	0.038	0.127	1.237	4.125	0.018	0.060	0.182	0.606	0.469	1.563	0.011	0.034
Chard for raw salads (*Beta vulgaris*)	OP	1.030	3.435	0.005	0.018	0.032	0.106	0.015	0.050	0.151	0.505	0.391	1.302	0.009	0.028
Cucumber (*Cucumis sativus*)	AB	0.400	1.335	0.002	0.007	0.012	0.041	0.006	0.019	0.059	0.196	0.152	0.506	0.004	0.011
	FP	0.605	2.017	0.003	0.011	0.019	0.062	0.009	0.029	0.089	0.296	0.229	0.765	0.006	0.017
Kiwi (*Actinidia deliciosa*)	NP	1.984	6.613	0.010	0.035	0.061	0.203	0.029	0.096	0.292	0.972	0.752	2.506	0.018	0.055
Lettuce (*Lactuca sativa*)	HP	0.005	0.018	0.032	0.106	1.034	3.446	0.015	0.050	0.152	0.506	0.392	1.306	0.009	0.028
Tomato (*Solanum lycopersicum*)	DB	0.767	2.558	0.004	0.013	0.024	0.079	0.011	0.037	0.113	0.376	0.291	0.969	0.007	0.021
	MP	0.801	2.670	0.004	0.014	0.025	0.082	0.012	0.039	0.118	0.392	0.304	1.012	0.007	0.022
	PB	0.576	1.920	0.003	0.010	0.018	0.059	0.008	0.028	0.085	0.282	0.218	0.728	0.005	0.016

Table S5

Analytical performance of the applied methodology for the analysis of heavy metals in soil and water.

	Linearity (R^2)	RSD (%)	LOD (mg/L)	LOQ (mg/L)	LOD (mg/kg dry weight)	LOQ (mg/kg dry weight)
Zn	0.9998	6.2	0.1090	0.3640	10.91	36.37
Cd	0.9990	19.4	0.0006	0.0019	0.06	0.19
Pb	0.9990	22.9	0.0034	0.0112	0.34	1.12
Ni	0.9992	18.0	0.0016	0.0053	0.16	0.54
Cu	0.9999	3.6	0.0160	0.0535	1.60	5.35

Cr	0.9999	5.6	0.0414	0.1379	4.14	13.79
Hg	0.9990	2.9	0.0010	0.0030	0.10	0.30

Table S6

Microbiological assessment of the food. Enumeration results were expressed in [log (CFU/g)]. (ND means that the parameter was not detected in the sample).

Type of food sample	Farmer ID	Microbiological parameters											
		E. coli	*Listeria* spp.	*B. cereus*	*Pseudomonas* spp.	Coagulase-positive Staphylococci	*C.perfringens*	*Enterobacteriaceae*	Yeasts	Molds	Aerobic mesophilic bacteria	Detection of *L. monocytogenes*	Detection of *Salmonella* spp.
Apple (*Malus domestica*)	BB	<1.0	<2.3	3.1±0.2	5.3±0.5	<2.3	<1.0	<1.0	<2.0	<2.0	4.5±0.1	ND	ND
	GP	<1.0	<2.3	<2.3	<2.3	<2.3	<1.0	<1.0	<2.0	<2.0	2.2±0.0	ND	ND
Bell pepper (*Capsicum annuum*)	CB	<1.0	<2.3	2.6±0.1	3.6±0.1	<2.3	<1.0	<1.0	<2.0	2.1±0.0	3.3±0.1	ND	ND
	EB	<1.0	<2.3	<2.3	3.9±0.3	<2.3	<1.0	<1.0	<2.0	<2.0	4.5±0.3	ND	ND
	IP	2.2±0.1	2.6±0.5	4.6±0.0	5.7±0.2	<2.3	<1.0	3.6±0.0	2.2±0.0	2.2±0.1	4.9±0.0	ND	ND
	JB	<1.0	<2.3	3.7±0.5	5.9±0.1	<2.3	<1.0	4.2±0.1	2.0±0.1	<2.0	>6.0	ND	ND
	QB	<1.0	<2.3	2.6±0.3	2.6±0.4	<2.3	<1.0	3.2±0.3	<2.0	<2.0	5.2±0.3	ND	ND
Bok choy (*Brassica rapa*)	KP	<1.0	<2.3	5.2±0.4	5.8±0.2	<2.3	<1.0	4.4±0.1	2.5±0.0	2.1±0.0	>6.0	ND	ND
Carrots (*Daucus	LP	<1.0	4.3±0.1	4.9±0.1	5.6±0.1	<2.3	1.8±0.1	3.5±0.0	<2.0	<2.0	>6.0	ND	ND

carota)													
Chard for raw salads (*Beta vulgaris*)	OP	<1.0	<2.3	5.2±0.01	5.6±0.0	<2.3	1.6±0.0	4.4±0.1	2.2±0.0	<2.0	>6.0	ND	ND
Cucumber	AB	<1.0	<2.3	3.2±0.2	4.8±0.5	<2.3	<1.0	<1.0	<2.0	<2.0	4.4±0.0	ND	ND
(*Cucumis sativus*)	FP	<1.0	<2.3	2.9±0.3	4.9±0.4	<2.3	<1.0	<1.0	<2.0	<2.0	3.4±0.0	ND	ND
Kiwi (*Actinidia deliciosa*)	NP	<1.0	<2.3	3.7±1.0	5.4±0.2	<2.3	<1.0	<1.0	<2.0	<2.0	3.7±0.1	ND	ND
Lettuce (*Lactuca sativa*)	HP	3.5±0.7	3.5±0.4	4.1±1.0	7.3±0.5	3.5±0.0	<1.0	6.0±0.0	2.8±0.0	2.2±0.0	>6.0	ND	ND
Tomato	DB	<1.0	<2.3	2.3±0.1	4.7±0.3	<2.3	<1.0	3.3±0.1	<2.0	<2.0	4.2±0.1	ND	ND
(*Solanum*	MP	<1.0	<2.3	<2.3	<2.3	<2.3	<1.0	<1.0	<2.0	<2.0	<1.0	ND	ND
lycopersicum)	PB	<1.0	<2.3	<2.3	5.8±0.2	<2.3	<1.0	5.4±0.1	<2.0	<2.0	>6.0	ND	ND

Table S7

Microbiological assessment of the soil. Enumeration results were expressed in [log (CFU/g)] (ND means that the parameter was not detected in the sample).

Farmer ID	Microbiological parameters							
	E. coli	*Listeria* spp.	*B. cereus*	*Pseudomonas* spp.	Coagulase-positive	*C. perfringens*	Detection of *L.*	Detection of

					Staphylococci		*monocytogenes*	*Salmonella* spp.
AB	3.7±0.6	4.9±0.5	5.2±0.5	5.9±0.1	4.1±0.5	3.2±0.0	ND	ND
BB	<1.0	4.0±0.2	3.7±1.2	5.3±0.0	3.4±0.2	3.7±0.1	ND	ND
CB	<1.0	4.9±0.3	4.5±1.0	2.9±1.0	3.8±0.6	2.9±0.1	ND	ND
DB	<1.0	5.0±0.2	4.3±0.8	4.6±0.9	3.8±0.2	4.2±0.0	ND	ND
EB	3.4±0.2	4.7±0.2	5.4±0.2	5.8±0.1	2.7±0.3	2.7±0.2	ND	ND
FP	3.2±0.1	4.9±0.2	<2.3	5.7±0.0	4.0±0.2	2.0±0.0	ND	ND
GP	2.9±0.0	4.2±0.1	4.8±2.2	5.9±0.1	2.9±0.5	2.8±0.0	ND	ND
HP	3.6±0.3	4.6±0.6	3.5±0.3	6.0±0.0	2.7±0.2	3.2±0.0	ND	ND
IP	<1.0	3.9±1.1	3.2±0.6	5.5±0.5	3.6±0.1	2.0±0.0	ND	ND
JB	<1.0	4.7±0.1	5.5±0.1	5.7±0.1	2.6±0.1	2.2±0.0	ND	ND
KP	<1.0	5.0±0.0	5.3±0.1	5.7±0.2	3.1±0.5	3.9±0.1	ND	ND
LP	<1.0	4.7±0.0	5.2±0.3	5.8±0.1	2.4±0.1	3.1±0.1	ND	ND
MP	<1.0	4.9±0.0	3.5±0.8	5.9±0.1	2.3±0.0	2.6±0.2	ND	ND
NP	5.4±0.1	4.4±0.1	5.1±0.2	5.5±0.1	2.4±0.1	2.3±0.1	ND	ND
OP	4.7±0.1	4.9±0.1	5.5±0.3	5.7±0.1	3.3±0.1	2.7±0.0	ND	ND
PB	3.2±0.3	4.7±0.1	5.3±0.0	5.9±0.1	3.8±0.2	3.7±0.1	ND	ND
QB	2.9±0.0	4.5±0.3	5.7±0.2	5.8±0.2	2.8±0.0	3.0±0.1	ND	ND

Table S8

Microbiological assessment of the irrigation water. Enumeration results were expressed in [log (CFU/l00 mL)] (ND means that the parameter was not detected in the sample and D means that the parameter was detected in the sample).

Farmer ID	Microbiological parameters											
	E. coli [log	*Listeria* spp. [log	*B. cereus*	*Pseudomonas* spp.	Coagulase-positive Staphylococci	*C. perfringens*	*Enterobacteriaceae*	Yeasts	Molds	Aerobic mesophilic bacteria	Detection of *L. monocytogenes*	Detection of *Salmonella* spp.
AB	<0.0	>2.0	>2.0	>2.0	<0.0	1.9±0.1	1.8±0.1	<0.0	<0.0	>2.0	ND	ND
BB	<0.0	<0.0	2.0±0.0	>2.0	<0.0	1.2±0.2	<0.00	<0.0	1.4±0.0	0.6±0.0	ND	ND
CB	<0.0	0.6±0.0	2.2±0.1	>2.0	<0.0	<0.0	<0.00	0.9±0.0	0.6±0.0	1.1±0.1	ND	ND
DB	<0.0	0.4±0.1	0.9±0.1	>2.0	<0.0	0.4±0.5	0.1±0.1	<0.0	1.4±0.0	0.9±0.0	ND	ND
EB	<0.0	<0.0	0.4±0.3	1.8±0.0	<0.0	0.2±0.2	<0.0	<0.0	1.2±0.1	>2.0	ND	ND
FP	<0.0	<0.0	0.9±0.4	1.6±0.0	<0.0	0.4±0.4	<0.0	<0.0	<0.0	1.9±0.0	**D**	ND
GP	1.5±0.1	<0.0	1.9±0.1	>2.0	<0.0	1.9±0.1	1.7±0.0	<0.0	2.0±0.0	1.9±0.0	**D**	ND
HP	0.6±0.3	<0.0	0.8±0.1	>2.0	<0.0	1.8±0.2	1.2±0.0	<0.0	1.7±0.0	>2.0	ND	ND
IP	<0.0	<0.0	0.3±0.2	1.7±0.0	<0.0	<0.0	<0.0	<0.0	0.9±0.0	0.6±0.0	ND	ND
JB	0.3±0.3	>2.0	0.4±0.3	>2.0	<0.0	>2.0	0.9±0.0	>2.0	<0.0	1.4±0.0	ND	ND
KP	<0.0	<0.0	>2.0	>2.0	<0.0	<0.0	<0.0	1.3±0.0	1.4±0.1	1.5±0.2	ND	ND
LP	<0.0	1.60±0.0	<0.0	>2.0	<0.0	0.1±0.1	<0.0	<0.0	0.6±0.1	>2.0	ND	ND
MP	<0.0	<0.0	>2.0	>2.0	<0.0	1.2±0.2	<0.0	1.7±0.1	1.7±0.1	1.9±0.1	ND	ND
NP	<0.0	<0.0	>2.0	1.9±0.3	<0.0	1.4±0.0	<0.0	<0.0	1.3±0.1	1.5±0.0	ND	ND
OP	0.8±0.2	0.9±0.3	>2.0	>2.0	<0.0	1.6±0.0	1.3±0.0	<0.0	<0.0	>2.0	ND	ND
PB	<0.0	>2.0	>2.0	>2.0	<0.0	1.2±0.1	<0.0	0.6±0.1	1.6±0.0	1.7±0.1	ND	ND
QB	<0.0	>2.0	>2.0	>2.0	>2.0	>2.0	<0.0	<0.0	0.6±0.0	>2.0	ND	ND

Table S9

Pesticide, PCBs and flame retardants concentrations found in farmers food. Concentration results expressed in µg/kg. Results above de LOQ are in bold.

Type of food sample	Farmer ID	Pesticide active compound	Results	MRLs	European regulation	Flame retardants /POP compound	Results
Apple (*Malus domestica*)	BB	Aldrin	0.10±0.02 (< LOD)	10	Reg. (EC) 839/2008	TBEP	**6.46±0.19 (> LOQ)**
		Cypermethrin	0.14±0.00 (< LOD)	10	Reg. (EU) 2017/626	TCP I	0.14±0.00 (< LOD)
		Deltamethrin I	0.15±0.02 (< LOD)	200	Reg. (EU) 2018/832		
	GP	Chlorpyrifos	**0.13±0.00 (> LOQ)**	10	Reg. (EU) 2020/1085	TBEP	**7.54±0.76 (> LOQ)**
		p,p´-DDD	**0.34±0.08 (> LOQ)**	50	Reg. (EU) 2023/163	TCP I	0.11±0.00 (< LOD)
Bell pepper (*Capsicum annuum*)	CB	Chlorfenvinphos	0.10±0.0 (< LOD)	10	Reg. (EU) 1138/2013	TBEP	**6.29±0.69 (> LOQ)**
		Chlorpyrifos	**0.14±0.01 (> LOQ)**	10	Reg. (EU) 2020/1085	TCP I	0.38 ±0.25 (< LOQ)
		Chlorpyrifos-methyl	0.24±0.00 (< LOQ)	10	Reg. (EU) 2020/1085	TCP II	0.10±0.00 (< LOD)
		p,p´-DDD	0.27±0.00 (< LOQ)	50	Reg. (EU) 2023/163	TEHP	**0.90±0.00(> LOQ)**
		Dimethoate	0.20±0.00 (< LOQ)	10	Reg. (EU) 2021/155		
	EB	---	---	---	---	TBEP	**6.58±0.99 (> LOQ)**
						TCP I	0.19±0.00 (< LOD)
	IP	Chlorpyrifos	**0.14±0.02 (> LOQ)**	10	Reg. (EU) 2020/1085	TBEP	**5.89±0.75 (> LOQ)**
		Dimethoate	0.19±0.00 (< LOQ)	10	Reg. (EU) 2021/155		
	JB	Chlorpyrifos	**0.13±0.00 (> LOQ)**	10	Reg. (EU) 2020/1085	TBEP	**6.37±0.66 (> LOQ)**
		p,p´-DDD	**0.58±0.00 (> LOQ)**	50	Reg. (EU) 2023/163	TCP I	0.13±0.00 (< LOD)
		Dimethoate	0.19±0.00 (< LOQ)	10	Reg. (EU) 2021/155		

	QB	p,p´-DDD	0.17±0.01 (< LOQ)	50	Reg. (EU) 2023/163	TBEP	**6.86±0.66 (> LOQ)**
Bok choy (*Brassica rapa*)	KP	---	---	---	---	TBEP	**6.93±0.47 (> LOQ)**
Carrots (*Daucus carota*)	LP	Bifenthrin	**3.12±1.53 (> LOQ)**	50	Reg. (EU) 2018/687	PCB153	1.16±0.00 (< LOQ)
		p,p´-DDD	0.19±0.02 (< LOQ)	50	Reg. (EU) 2023/163	TBEP	**6.37±0.33 (> LOQ)**
		Dieldrin	0.26±0.01 (< LOD)	10	Reg. (EU) 2015/399	TCPI	0.27±0.00 (< LOQ)
						TEHP	**0.87±0.00 (> LOQ)**
Chard for raw salads (*Beta vulgaris*)	OP	---	---	---	---	TBEP	**7.99±1.04 (> LOQ)**
						TCP I	0.42±0.00 (< LOQ)
Cucumber (*Cucumis sativus*)	AB	Chlorpyrifos-methyl	0.25±0.00 (< LOQ)	10	Reg. (EU) 2020/1085	TBEP	**7.29±0.84 (> LOQ)**
		p,p´-DDD	0.44±0.06 (< LOQ)	50	Reg. (EU) 2023/163	TCP I	0.10±0.00 (< LOD)
						TCP III	0.16±0.00 (< LOD)
	FP	Chlorpyrifos	**0.13±0.00 (> LOQ)**	10	Reg. (EU) 2020/1085	TBEP	**5.81±3.09 (> LOQ)**
		p,p´-DDD	**0.89±0.00 (> LOQ)**	50	Reg. (EU) 2023/163	TEHP	**0.91±0.00 (> LOQ)**
Kiwi (*Actinidia deliciosa*)	NP	Chlorpyrifos-methyl	0.24±0.00 (< LOQ)	10	Reg. (EU) 2020/1085	TBEP	**7.47±0.95 (> LOQ)**
		p,p´-DDD	0.50±0.21 (< LOQ)	50	Reg. (EU) 2023/163		
Lettuce (*Lactuca sativa*)	HP	Chlorpyrifos	**0.13±0.00 (> LOQ)**	10	Reg. (EU) 2020/1085	TBEP	**6.64±0.00 (> LOQ)**
		Dieldrin	0.24±0.00 (< LOD)	10	Reg. (EC) 839/2008		
						TEHP	**0.85±0.00 (> LOQ)**
Tomato (*Solanum lycopersicum*)	DB	p,p´-DDD	0.18±0.02 (< LOQ)	50	Reg. (EU) 2023/163	TBEP	**8.15±0.76 (> LOQ)**
		Dieldrin	0.23±0.00 (< LOD)	10	Reg. (EC) 839/2008		
	MP	---	---	---	---	TBEP	**5.42±0.12 (> LOQ)**
						TCP I	0.16±0.00 (< LOD)

PB	p,p´-DDD	**0.71±0.00 (> LOQ)**	50	Reg. (EU) 2023/163	TBEP	**5.79±1.17 (> LOQ)**
	Dimethoate	0.19±0.00 (< LOQ)	10	Reg. (EU) 2021/155	TCP I	0.11±0.00 (< LOD)

Table S10

Pesticide, PCBs and flame retardants concentrations found in farmer's soil. Concentration results expressed in µg/kg. Results above de LOQ are in bold.

Farmer ID	Agrochemical active compound	Results	Maximum limit	Flame retardants/POP compound	Results
AB	Chlorpyrifos	**0.15±0.02 (> LOQ)**		TBEP	**6.52±0.47 (> LOQ)**
	p,p´-DDD	**1.09 ±0.00 (> LOQ)**	100 [a]	TCP I	0.32±0.24 (< LOQ)
BB	Chlorpyrifos-methyl	0.14±0.15 (< LOQ)		TBEP	**5.19±0.57 (> LOQ)**
	p,p´-DDD	**0.70±0.48 (> LOQ)**	100 [a]		
	Dieldrin	0.25±0.00 (< LOD)	50 [a]		
	Dimethoate	0.19±0.00 (< LOQ)			
	Endosulfan I	**1.59±0.00 (> LOQ)**	10 [a] /40 [b]		
CB	Aldrin	**10.26 ±0.78 (> LOQ)**	50 [a]	TBEP	**6.00±0.57 (> LOQ)**
	Chlorpyrifos	**0.31±0.03 (> LOQ)**		TCP I	0.13±0.04 (< LOD)
	Chlorpyrifos-methyl	0.24 ±0.00 (< LOQ)		TCP II	0.36±0.16 (< LOD)
	Dimethoate	0.19±0.00 (< LOQ)			
DB	Chlorpyrifos	**0.13±0.01 (> LOQ)**		TBEP	**7.46±0.70 (> LOQ)**
	p,p´-DDD	0.50±0.30 (< LOQ)	100 [a]		
	Dimethoate	0.19±0.00 (< LOQ)			
EB	Chlorpyrifos-methyl	0.24±0.00 (< LOQ)		TBEP	**5.32±0.66 (> LOQ)**

				TCP I	0.24±0.00 (< LOD)
FP	---	---	---	TBEP	**7.07±0.01 (> LOQ)**
				PCB180	0.35±0.00 (< LOD)
GP	Chlorpyrifos	**0.13±0.00 (> LOQ)**		TBEP	**6.96±0.80 (> LOQ)**
				TCP I	**0.96±0.43 (> LOQ)**
HP	---	---		TBEP	**6.84±0.99 (> LOQ)**
IP	Chlorpyrifos	**0.66±0.46 (> LOQ)**		TBEP	**4.64±3.67 (> LOQ)**
	Chlorpyrifos-methyl	0.25±0.00 (< LOQ)			
	p,p´-DDD	0.52±0.15 (< LOQ)	100 [a]		
	Dimethoate	0.19±0.00 (< LOQ)			
JB	Chlorpyrifos	**0.13±0.00 (> LOQ)**		TBEP	**7.67±1.15 (> LOQ)**
	p,p´-DDD	0.31±0.16 (< LOQ)	100 [a]	TCP I	0.12±0.02 (< LOD)
KP	Aldrin	1.25±0.85 (< LOQ)	50 [a]	TBEP	6.39±0.49 **(> LOQ)**
	Chlorpyrifos	**0.13±0.00 (> LOQ)**		TCP I	0.28±0.00 (< LOQ)
				TnBP	**0.85±0.00 (> LOQ)**
LP	Chlorpyrifos	**0.13±0.00 (> LOQ)**		TBEP	**4.19±0.72 (> LOQ)**
	Cyhalothrin II	0.59±0.00 (< LOQ)			
	p,p´-DDD	0.44±0.21 (< LOQ)	100 [a]		
MP	p,p´-DDD	0.51±0.53 (< LOQ)	100 [a]	TBEP	**5.09±0.57 (> LOQ)**
				TCP I	0.43±0.00 (< LOQ)
NP	Cholfenvinphos	0.12±0.00 (< LOD)		TBEP	**6.17±0.71 (> LOQ)**
	p,p´-DDD	**0.68±0.38 (> LOQ)**	100 [a]		
	p,p´-DDE	0.12±0.00 (< LOD)	100 [a]		

OP	Chorpyrifos	**2.81±3.41 (> LOQ)**		TBEP	**5.85±0.63 (> LOQ)**
	p,p´-DDD	0.47±0.48 (< LOQ)	100 [a]	TCP II	0.11±0.00 (< LOD)
PB	Chlorpyrifos	**0.13±0.00 (> LOQ)**		TBEP	**7.30±0.51 (> LOQ)**
	p,p´-DDD	0.17±0.00 (< LOQ)	100 [a]	TCP I	0.21±0.00 (< LOD)
QB	Chlorpyrifos	0.12±0.00 (= LOQ)		TBEP	**7.45±1.45 (> LOQ)**

a Reference values from Ministry of the Environment, Finland. Government Decree on the Assessment of Soil Contamination and Remediation Needs (214/2007)

b Reference values from APA, 2022

Table S11

Pesticide, PCBs and flame retardants concentrations found in farmers irrigation water. Concentration results expressed in µg/L. Results equals and above the LOQ are in bold.

Farmer ID	Agrochemical active compound	LOD (fd 250x)	LOQ (fd 250x)	Results	Flame retardants/POP compound	LOD (fd 250x)	LOQ (fd 250x)	Results
AB	Chorpyrifos	0.005	0.016	**0.020±0.289 (> LOQ)**	--	--	--	--
	Chorpyrifos-methyl	0.015	0.049	0.032±0.000 (<LOQ)				
BB	Chorpyrifos	0.005	0.016	**0.017±0.016 (> LOQ)**	--	--	--	--
	Chorpyrifos-methyl	0.015	0.049	0.032±0.000 (<LOQ)				
CB	Chorpyrifos-methyl	0.015	0.049	0.032±0.000 (<LOQ)	--	--	--	--
	p,p´-DDD	0.021	0.070	0.028±0.010 (<LOQ)				
EB	Dimethoate	0.022	0.075	0.026±0.000 (<LOQ)	--	--	--	--
GP	Chlorpyrifos	0.005	0.016	**0.017±0.000 (> LOQ)**	--	--	--	--
	p,p´-DDD	0.021	0.070	0.028±0.000 (<LOQ)				
HP	Chorpyrifos-methyl	0.015	0.049	0.032±0.000 (<LOQ)	--	--	--	--
JB	Bifentrin	0.037	0.122	**0.407±1.937 (> LOQ)**	--	--	--	--

KP	Bifentrin	0.037	0.122	**0.213±0.000 (> LOQ)**	--			--
	Chorpyrifos	0.005	0.016	**0.016±0.000 (> LOQ)**				
LP	Chorpyrifos	0.005	0.016	**0.016±0.000 (> LOQ)**	TCP III	0.064	0.213	**0.467±0.000 (> LOQ)**
	Chorpyrifos-methyl	0.015	0.049	0.032±0.000 (<LOQ)				
NP	Chorpyrifos	0.005	0.016	**0.018±0.000 (> LOQ)**	--			--
OP	--	--	--	--	TCP III	0.064	0.213	**2.601±0.000 (> LOQ)**
PB	p,p´-DDD	0.021	0.070	0.034±0.000 (<LOQ)	--	--	--	--
QB	Chorpyrifos-methyl	0.015	0.049	0.034±0.000 (<LOQ)	--	--	--	--

Table S12

Nitrate concentrations found in farmers food, soil and water. Results above the established limits are in bold.

Type of food sample	Farmer ID	Food (mg/kg)	Soil (mg/kg)	Water (mg/L)
Apple (*Malus domestica*)	BB	11±3	**64±10**	**52±10**
	GP	16±1	**55±6**	13±4
Bell pepper (*Capsicum annuum*)	CB	23±6	**3084±234**	5±0
	EB	45±13	**79±14**	1±0 (< LOQ)
	IP	11±4	**200±32**	46±13
	JB	43±10	**214±21**	**76±17**
	QB	17±4	**97±13**	4±2
Bok choy (*Brassica rapa*)	KP	**1079±100**	**268±32**	--
Carrots (*Daucus carota*)	LP	**825±32**	50±11	13±6
Chard for raw salads (*Beta vulgaris*)	OP	**2599±164**	**1661±156**	--

Cucumber (*Cucumis sativus*)	AB	111±21	**71±12**	5±1
	FP	90±10	**1421±146**	12±5
Kiwi (*Actinidia deliciosa*)	NP	9±2	**52±8**	1±0 (< LOQ)
Lettuce (*Lactuca sativa*)	HP	**1484±146**	**588±101**	11±3
Tomato (*Solanum lycopersicum*)	DB	**124±15**	**90±12**	30±6
	MP	34±1	**85±7**	--
	PB	**100±26**	48±4	2±0

Table S13

Heavy metals found in farmers food in mg/kg of fresh weight. Results above the LOQ are in bold.

Type of sample	Farmer ID	Zn	Cd	Pb	Ni	Cu	Cr	Hg
Apple (*Malus domestica*)	BB	1.842±0.521 (<LOQ)	0.019±0.003 (<LOQ)	0.045±0.022 (<LOQ)	--	**1.506±0.621**	0.010±0.004 (<LOD)	--
	GP	0.180±0.061 (<LOD)	0.024±0.010 (<LOQ)	0.040±0.012 (<LOD)	0.014±0.007 (<LOD)	0.179±0.043 (<LOD)	0.015±0.004 (<LOD)	--
Bell pepper (*Capsicum annuum*)	CB	1.956±0.201 (<LOQ)	**0.023±0.009**	0.016±0.006 (<LOD)	0.009±0.002 (<LOD)	**0.961±0.092**	0.010±0.000 (<LOD)	--
	EB	1.461±0.312 (<LOQ)	**0.017±0.005**	0.037±0.014 (<LOQ)	0.004±0.000 (<LOD)	**0.729±0.027**	0.018 ±0.006(<LOD)	--
	IP	1.475±0.256 (<LOD)	0.008±0.002 (<LOD)	0.024±0.007 (<LOQ)	0.024±0.010 (<LOQ)	**0.692±0.143**	0.007±0.003 (<LOD)	--
	JB	1.028±0.712 (<LOQ)	0.008±0.003 (<LOQ)	0.013±0.005 (<LOD)	**0.024±0.015**	**0.567±0.076**	0.014±0.006 (<LOD)	--
	QB	2.269±0.731 (<LOQ)	**0.019±0.011**	0.012±0.005 (<LOD)	0.020 (<LOQ) ±0.011	**0.993±0.134**	0.022±0.011 (<LOD)	--
Bok choy (*Brassica rapa*)	KP	**5.422±1.054**	**0.027±0.006**	**0.093±0.025**	**0.051±0.010**	0.286±0.065 (<LOQ)	0.182±0.071 (<LOD)	--
Carrots (*Daucus carota*)	LP	**6.529±1.328**	**0.054±0.015**	**0.156±0.043**	**0.069±0.035**	**0.966±0.365**	0.104±0.052 (LOQ)	--
Chard for raw salads (*Beta vulgaris*)	OP	**4.608±1.612**	**0.084±0.032**	**0.450±0.126**	**0.278±0.127**	1.507±0.719 (<LOD)	**1.762±0.582**	--

Cucumber (*Cucumis sativus*)	AB	**1.699±0.412**	0.006±0.000 (<LOQ)	0.015±0.003 (<LOQ)	0.017±0.009 (<LOQ)	**0.313±0.062**	0.006±0.002 (<LOD)	--
	FP	**2.179±0.841**	0.010±0.006 (<LOQ)	0.017±0.006 (<LOD)	0.013±0.004 (<LOQ)	**0.341±0.067**	0.008±0.004 (<LOD)	--
Kiwi (*Actinidia deliciosa*)	NP	1.276±0.775 (<LOD)	0.023±0.011 (<LOQ)	0.028±0.010 (<LOD)	0.062±0.020 (<LOQ)	**1.523±0.347**	--	--
Lettuce (*Lactuca sativa*)	HP	**5.860±2.634**	0.060±0.032 (<LOQ)	0.033±0.015 (<LOQ)	0.042±0.026 (<LOQ)	**0.752±0.111**	0.051±0.016 (<LOD)	--
Tomato (*Solanum lycopersicum*)	DB	**2.743±0.859**	**0.015±0.009**	0.023±0.011 (<LOD)	--	**0.545±0.120**	0.007±0.004 (<LOD)	--
	MP	1.371±0.923 (<LOQ)	**0.026±0.015**	0.029±0.006 (<LOQ)	0.001±0.000 (<LOD)	**0.425±0.116**	0.011±0.005 (<LOD)	--
	PB	1.642±0.428 (<LOQ)	**0.017±0.008**	0.011±0.005 (<LOD)	0.010±0.005 (<LOQ)	**0.547±0.256**	0.006±0.001 (<LOD)	--

Table S14

Heavy metals found in farmers' soil (mg/kg dry weight). Results equals and above the LOQ are in bold.

Farmers' ID	Zn	Cd	Pb	Ni	Cu	Cr	Hg
AB	22.85±1.24 (<LOQ)	**0.61±0.25**	**2.88±0.46**	**4.56±3.12**	**35.85±3.00**	**34.94±2.33**	--
BB	--	0.17±0.08 (<LOQ)	1.03±0.83 (<LOQ)	--	--	--	--
CB	14.57±0.93 (<LOQ)	**0.35±0.12**	**5.10±3.46**	**2.18±0.24**	**29.95±4.00**	**19.03±5.54**	--
DB	19.12±1.33 (<LOQ)	**0.43±0.18**	**4.56±2.82**	**2.95±1.85**	**30.19±1.42**	**22.12±0.38**	--
EB	25.90±3.44 (<LOQ)	**0.33±0.17**	**4.48±3.15**	**5.03±3.25**	**24.58±1.38**	**30.53±1.62**	--
FP	**37.13±1.47**	**0.67±0.31**	**12.52±10.72**	**18.33±0.50**	**41.46±3.21**	**61.49±4.10**	--
GP	18.35±2.79 (<LOQ)	**0.56±0.20**	**3.78±3.00**	**4.29±1.57**	**72.72±2.00**	**35.22±2.11**	--
HP	23.12±0.47 (<LOQ)	**0.64±0.16**	**4.01±2.37**	**8.36±3.78**	**27.96±1.02**	**40.30±5.26**	--
IP	18.00±1.77 (<LOQ)	**0.85±0.26**	**6.57±3.47**	**3.66±2.48**	**46.33±6.93**	**41.05±2.29**	--
JB	15.10±4.00 (<LOQ)	**0.27±0.03**	**1.35±0.64**	**3.37±0.15**	**18.05±2.00**	**31.99±4.63**	--
KP	10.24±4.33 (<LOD)	0.13±0.08 (<LOQ)	**1.70±1.23**	**2.26±0.92**	1.08±0.082 (<LOD)	**14.52±1.18**	--

LP	--	0.08±0.05 (<LOQ)	0.89±0.82 (<LOQ)	--	--	--	--
MP	17.07±0.26 (<LOQ)	**0.22±0.14**	**2.68±1.85**	**2.74±2.10**	**30.66±4.00**	**16.27±1.58**	--
NP	18.20±0.38 (<LOQ)	**0.92±0.52**	**8.06±2.53**	**10.19±1.85**	**21.00±2.46**	**58.97±2.50**	--
OP	26.62±0.67 (<LOQ)	**0.60±0.25**	**3.29±2.55**	**9.36±1.72**	**44.78±3.73**	**63.02±1.56**	--
PB	34.76±0.67 (<LOQ)	0.18±0.08 (<LOQ)	**4.66±2.37**	**2.71±1.92**	**21.81±1.93**	**21.62±2.04**	--
QB	34.21±1.00 (<LOQ)	**0.61±0.48**	**5.79±4.90**	**7.49±1.53**	**29.49±7.46**	**44.23±2.38**	--

Table S15

Heavy metals found in irrigation water (mg/L). Results above the LOQ are in bold.

Farmers' ID	Zn	Cd	Pb	Ni	Cu	Cr	Hg
AB	--	0.0003±0.0004 (<LOD)	0.0024±0.0008 (<LOD)	--	--	0.0008±0.0003 (<LOD)	--
BB	0.0280±0.014 (<LOD)	--	--	--	--	--	--
CB	--	0.0006±0.0010 (<LOD)	0.0041±0.0021 (<LOQ)	--	--	0.0007±0.0003 (<LOD)	--
DB	0.0173±0.0045 (<LOD)	0.0005±0.0001 (<LOD)	**0.0218±0.0073**	0.0001±0.0000 (<LOD)	--	0.0005±0.0003 (<LOD)	--
EB	**0.2503±0.0075**	0.0007±0.0001 (<LOD)	0.0027±0.0012 (<LOD)	0.0037±0.0010 (<LOQ)	0.0050±0.0021 (<LOD)	0.0002±0.0000 (<LOD)	--
FP	--	0.0003±0.005 (<LOD)	0.0040±0.0012 (<LOQ)	0.0005±0.0003 (<LOD)	--	0.0010±0.0007 (<LOD)	--
GP	--	0.0003±0.0001 (<LOD)	0.0009±0.0010 (<LOD)	--	--	--	--
HP	--	0.0003±0.0001 (<LOD)	0.0029±0.0016 (=LOD)	--	0.0015±0.0009 (<LOD)	0.0006±0.002 (<LOD)	--
IP	--	0.0004±0.005 (<LOD)	0.0022±0.0014 (<LOD)	--	--	--	--
JB	0.0123±0.0054 (<LOD)	0.0008±0.0003 (<LOQ)	0.0043±0.0011 (<LOQ)	0.0007±0.0001 (<LOD)	--	--	--
KP	--	0.0004±0.0006 (<LOD)	0.0029±0.0007 (=LOD)	0.0017±0.0006 (<LOD)	--	--	--
LP	--	0.0009±0.0006 (<LOD)	0.0042±0.0010 (<LOQ)	--	--	0.0002±0.0002 (<LOD)	--

MP	--	0.0005±0.0001 (<LOD)	0.0025±0.0007 (<LOQ)	0.0006±0.0003 (<LOD)	--	0.0004±0.0000 (<LOD)	--
NP	--	0.0005±0.0002 (<LOD)	0.0022±0.0006 (<LOD)	0.0043±30.0015 (<LOQ)	--	0.0009±0.0005 (<LOD)	--
OP	--	0.0004±0.0002 (<LOD)	0.0105±0.0090 (<LOQ)	--	--	0.0007±0.0002 (<LOD)	--
PB	--	0.0004±0.0001 (<LOD)	0.0027±0.0005 (<LOD)	--	0.0009±0.0012 (<LOD)	--	--
QB	--	0.0005±0.0006 (<LOD)	0.0013±0.0005 (<LOD)	0.0005±0.0005 (<LOD)	--	0.0002±0.0000 (<LOD)	--
